# Diagnostic and prognostic value of miR-146b-5p in acute pancreatitis

**DOI:** 10.1186/s41065-025-00466-9

**Published:** 2025-05-31

**Authors:** Ying Liao, Weiwei Zhang, Zhenfei Huang, Liu Yang, Mingjin Lu

**Affiliations:** 1 Department of Critical Care Medicine, Ganzhou People′s Hospital, Ganzhou City, Jiangxi Province 341100 China; 2 Supply Room, The Fifth People’s Hospital of Ganzhou, No. 666, Dongjiangyuan Avenue, Shuixi Town, Zhanggong District, Ganzhou City, Jiangxi Province 341000 China

**Keywords:** Acute pancreatitis, miR-146b-5p, DCA, Biomarker

## Abstract

**Objective:**

MicroRNAs hold great potential as biomarkers for assessing the progression of acute pancreatitis (AP). This study aimed to explore the value of miR-146b-5p in the diagnosis and prognosis of AP patients.

**Methods:**

110 AP patients were included and divided into 40 severe AP (SAP) patients and 70 non-SAP patients based on disease severity. Serum miR-146b-5p levels were measured using RT-qPCR. The diagnostic value of miR-146b-5p was evaluated utilizing ROC curves. Pearson correlation coefficient was employed to analyze the correlations between APACHEII, BISAP, and MCTSI scores and miR-146b-5p levels. The AP cell model was constructed by treating AR42J cells with deoxycholic acid (DCA), the proliferative capacity of cells was measured with CCK-8, apoptosis was measured by flow cytometry, and IL-6 and IL-8 protein levels were analyzed by ELISA.

**Results:**

Serum miR-146b-5p levels were decreased in SAP and unfavorable patients. Serum miR-146b-5p was able to effectively differentiate between SAP and non-SAP patients, and also effectively differentiate between unfavorable and favorable patients. MiR-146b-5p levels were significantly negatively correlated with APACHEII score (*r*=-0.6676), BISAP score (*r*=-0.5696), and MCTSI score (*r*=-0.5857). Furthermore, in the AP cell model, miR-146b-5p expression was down-regulated, proliferative capacity was diminished, apoptosis was increased, and IL-6 and IL-8 levels were elevated, but overexpression of miR-146b-5p partially reversed these changes.

**Conclusion:**

miR-146b-5p expression is down-regulated in the serum of SAP patients and cells, and it has a good diagnostic effect. It may be a potential biomarker and therapeutic target for AP.

**Supplementary Information:**

The online version contains supplementary material available at 10.1186/s41065-025-00466-9.

## Introduction

Acute pancreatitis (AP) represents a frequently encountered acute and severe pancreatic disorder in clinical practice [1]. It is distinguished by its abrupt commencement, swift progression of the pathological condition, a multitude of associated complications, and a relatively high fatality rate [2]. Currently, in the clinical field, the diagnosis of AP primarily relies on blood and urine-related examinations and imaging tests [3]. Therefore, it is important to explore novel biomarkers and molecular targets to enhance the prognosis of patients with AP.

MicroRNAs (miRNAs) are tiny and highly conserved non-coding RNA molecules [4]. They can bind to mRNA through translation inhibition or mRNA degradation pathways and can participate in the post-transcriptional regulatory process [5]. Numerous studies have demonstrated that miRNAs play a significant role in the development and progression of diseases [6]. Hassan et al. noted that in vitro knockdown of miR-155 inhibited leukemia cells and HCV viral loads [7]. El-Khazragy et al. revealed an association between circulating miRNAs and tissue iron overload [8]. Selvakumar et al. also found that in preeclampsia conditions, miR-510-3p could modulate vascular dysfunction by targeting VEGFA and its signaling axis [9]. These studies further confirm the important impact of miRNAs in different disease processes. As research progresses, new breakthroughs in the use of non-coding RNAs for disease treatment keep emerging [10]. Abaza et al. found that cyclic RNAs are potentially valuable in hepatocellular carcinoma immunotherapy, providing new perspectives on the study of non-coding RNAs for the treatment of disease [11]. Recent studies have also indicated that miRNAs can be consistently detected in serum and they have the potential to be markers for disease diagnosis and prognosis [12]. Notably, Liu et al. demonstrated through microarray analysis that miR-146b-5p was downregulated in the serum of AP patients [13]. Nonetheless, the investigation did not perform a detailed study on miR-146b-5p. In retrospect, early studies have focused on the field of oncology, and relatively little attention has been paid to diseases such as AP [14]. In addition, the limited technical conditions at the early stage and the complexity of AP pathogenesis have made it difficult to conduct in-depth studies on miR-146b-5p in AP, and the related explorations are still insufficient [15].

In this study, serum miR-146b-5p was clinically examined in AP patients with the aim of clarifying its diagnostic value in AP and constructing an in vitro cell model to explore its function. The study analyzed the potential of miR-146b-5p as a diagnostic and prognostic biomarker of AP, and deeply analyzed its regulatory role in the pathogenesis of AP. At the same time, the possibility of miR-146b-5p as a potential therapeutic target for AP was explored, and these findings are expected to open a new path for basic research and clinical practice of AP.

## Method and materials

### Sample selection

The Ethics Committee of *The Fifth People’s Hospital of Ganzhou* approved the study. The approval date was 2022-03-14, and the approval number was No. 20,220,920. All subjects gave full informed consent. Initial planning was performed with reference to previous biomarker studies and hospital AP admissions, taking into account practical constraints such as patient recruitment time, cost, and experimental resources [16–18]. The inclusion and exclusion criteria were strictly enforced to ensure the quality of the samples, and a total of 110 AP patients were recruited into the study. Inclusion criteria for AP patients: (1) diagnostic criteria for AP are defined based on the Atlanta classification [19]: I. The clinical manifestations are typically acute and persistent abdominal pain, II. The serum amylase or lipase levels exceed 3 times the upper limit of normal value, III. The CT, abdominal ultrasound and other imaging findings showing features of AP, (2) first onset of illness and admission time < 48 h, (3) no use of glucocorticoids or immunosuppressants, (4) complete clinical data. The severity of AP was assessed according to the Atlanta criteria and categorized as mild, moderate, and severe [19]. Mild AP was defined as the absence of organ failure (OF) and local or systemic complications. Moderate-to-severe AP was defined as temporary OF that dissipated within 48 h, accompanied by local or systemic complications. SAP was defined as unremitting OF. According to the severity of the disease, AP patients were categorized into SAP group (*n* = 40) and non-SAP group (*n* = 70). When analyzed by G*Power software, the Power value was 0.81, indicating that the efficacy of the statistical test for the sample size of this study was good. In addition, the acute physiology and chronic health evaluation II (APACHE II), bedside index of severity in acute pancreatitis (BISAP), and modified computed tomography severity index (MCTSI) scoring systems were used in the study. Clinical data such as age, gender, body mass index (BMI), etiology, white blood cell count (WBC), hemoglobin (HB), hematocrit (HCT), C-reaction protein (CRP), interleukin 6 (IL-6) and IL-8 were gathered from AP patients and are enumerated in Table 1. A list of article abbreviations is added to Table S1. All subjects fasted for 12 h before treatment and blood was gathered in a fasting state on the subsequent day. The collected blood was separated and serum was extracted and preserved at -80℃.

**Table 1:** The clinical information comparisons of AP patients in different severity

Variables	Non-SAP (*n* = 70)	SAP (*n* = 40)	*P* values
Age (years)	50.86±8.22	52.15±8.39	0.433
Gender (male/female)	39/31	23/17	0.856
BMI (kg/m^2^)	23.18±2.47	22.35±2.80	0.109
Etiology (n)			
Biliary	22	10	0.255
Alcohol	3	5	
Hyperlipidemia	45	25	
WBC (×10^9^/L)	11.73±3.31	12.72±2.45	0.102
HB (g/L)	144.03±16.13	141.31±26.12	0.501
HCT (%)	41.68±2.23	41.70±0.91	0.941
CRP (mmol/L)	96.47±17.53	228.30±35.63	< 0.001
IL-6 (pg/mL)	29.56±2.96	64.84±15.36	< 0.001
IL-8 (pg/mL)	27.55±3.63	42.26±5.90	< 0.001
APACHEII score	5.97±0.85	11.40±1.45	< 0.001
BISAP score	2.01±0.47	3.73±0.75	< 0.001
MCTSI score	3.07±1.05	4.60±1.69	< 0.001

Abbreviations: WBC = white blood cell count; HB = hemoglobin; HCT = hematocrit; AMY = serum amylase; LPS = lipase; IL-6 = interleukin 6; CRP = C-reaction protein; APACHEII = acute physiology and chronic health evaluation II, MCTSI = Modified computed tomography severity index; BISAP = bedside index of severity in acute pancreatitis; *P* < 0.05 means significant difference

### Prognosis

Patients were tracked for 28 days after their release from the hospital and were sorted into a favorable prognosis category (*n* = 94) and an unfavorable prognosis category (*n* = 16) depending on whether secondary infections in the abdominal region occurred, organ failure occurred, or death occurred.

### Real-time quantitative PCR(RT-qPCR)

Total RNA was isolated from serum samples using TRIzol reagent (Thermo Fisher Scientific, USA.) according to the manufacturer’s instructions. A PrimeScript RT kit (TaKaRa, Japan) was utilized to synthesize the cDNA. The synthesized cDNA was amplified using SYBR Green Real-time PCR Master Mix (Toyobo, Japan). U6 served as the internal control, and the relative expression level of miR-146b-5p was determined in accordance with Eq. 2^−ΔΔCt^.

### Cell culture, transfection and AP model construction

Rat pancreatic exocrine cells (AR42J) are able to better mimic the physiological functions of pancreatic cells and their response to injury, and are a commonly used cell line for constructing AP in vitro models [20–22]. Therefore, AR42J cells were selected for the study to construct the AP model. AR42J cells were grown in F-12 K medium (Sigma-Aldrich, USA) containing 10% FBS (Sigma-Aldrich) and 1% penicillin-streptomycin (Gibco, USA). The cells were incubated in an incubator at 37 °C under 5% CO_2_. AR42J cells were initially cultured in 6-well plates prior to the experiment. When the cell density reached 80%, the cells were incubated with 0.4 mmol/L DCA (Sigma-Aldrich) for 30 min to construct the AP cell model [23]. The control group consisted of untreated AR42J cells. The transfection of cells was carried out using Lipofectamine 2000 reagent (Invitrogen, USA) following the manufacturer’s instructions. All subsequent experiments were conducted on the basis of cultured and established AP cell models.

### Cell proliferation

Cell Counting Kit-8 (Beyotime, Shanghai) was employed to determine how miR-146b-5p affects the proliferative potential of DCA-treated AR42J cells. Cells in good growth condition were resuspended, and then inoculated into 96-well plates at a density of 1 × 10^4^ cells/well. Subsequently, plasmids were transfected into the cells respectively. At 0, 1, 2, 3, and 4 days after transfection, 10µL of CCK-8 reagent was put into the 96-well plate and incubated within the incubator for 1 h, respectively. Cell viability was measured using a microplate reader.

### Cell apoptosis

To assess how miR-146b-5p affects apoptosis in DCA-treated AR42J cells, Annexin V-FITC Apoptosis Detection Kit (Beyotime) was used. Cells in favorable growth conditions were seeded into 6-well plates at a cell density of 2 × 10^5^ cells/well. Then, plasmids were transfected into the cells respectively. 24 h post-transfection, the cells were harvested and washed with PBS. Then, AV and PI dyes were added respectively, and the mixture was incubated in the dark for 30 min. After that, the cell suspension was filtered through a membrane and assayed by flow cytometer.

### Enzyme-linked immunosorbent assay (ELISA)

The ELISA Kit (Thermo Fisher Scientific) was used to detect the protein levels of inflammatory cytokines. After DCA treatment, the cells were harvested, and IL-6 and IL-8 levels were determined respectively in accordance with the manufacturer’s instructions.

### Statistical analysis

The data were analyzed using GraphPad Prism 9.0 (GraphPad Software, Inc., USA) and SPSS 23.0 (SPSS, Inc., Chicago, USA) software. Mean ± standard deviation (SD) was adopted to express the data. One-way ANOVA was used for comparisons between multiple groups, and Tukey’s test was used for multiple comparisons between groups, t-test was used for comparisons between two groups. The diagnostic value of miR-146b-5p for AP was determined by ROC curve analysis. The Shapiro-Wilk test was used to evaluate the normality of serum miR-146b-5p level, APACHE II score, BISAP score and MCTSI score. Pearson correlation analysis was performed for those conforming to the normal distribution, while Spearman rank correlation analysis was used as a supplement for non-normal distribution variables. The factors that affected the poor prognosis of SAP patients were analyzed by means of logistic regression. *P* < 0.05 was deemed to denote a statistically significant difference.

## Results

### Basic information of all subjects

The results of the statistical analysis in Table 1 showed that among all subjects, the differences in age, gender, BMI, etiology, WBC, HB and HCT indicators were not statistically significant between the SAP and non-SAP groups (*P* > 0.05). However, in CRP, IL-6 and IL-8 indexes, there were significant differences between two groups (*P* < 0.001). In addition, the APACHEII score, BISAP score and MCTSI score in the SAP group showed statistically significant differences compared with the corresponding three scoring indicators in the non-SAP group (*P* < 0.001).

### miR-146b-5p is downregulated and has high diagnostic value in serum of SAP and unfavorable patients

**Fig. 1:**
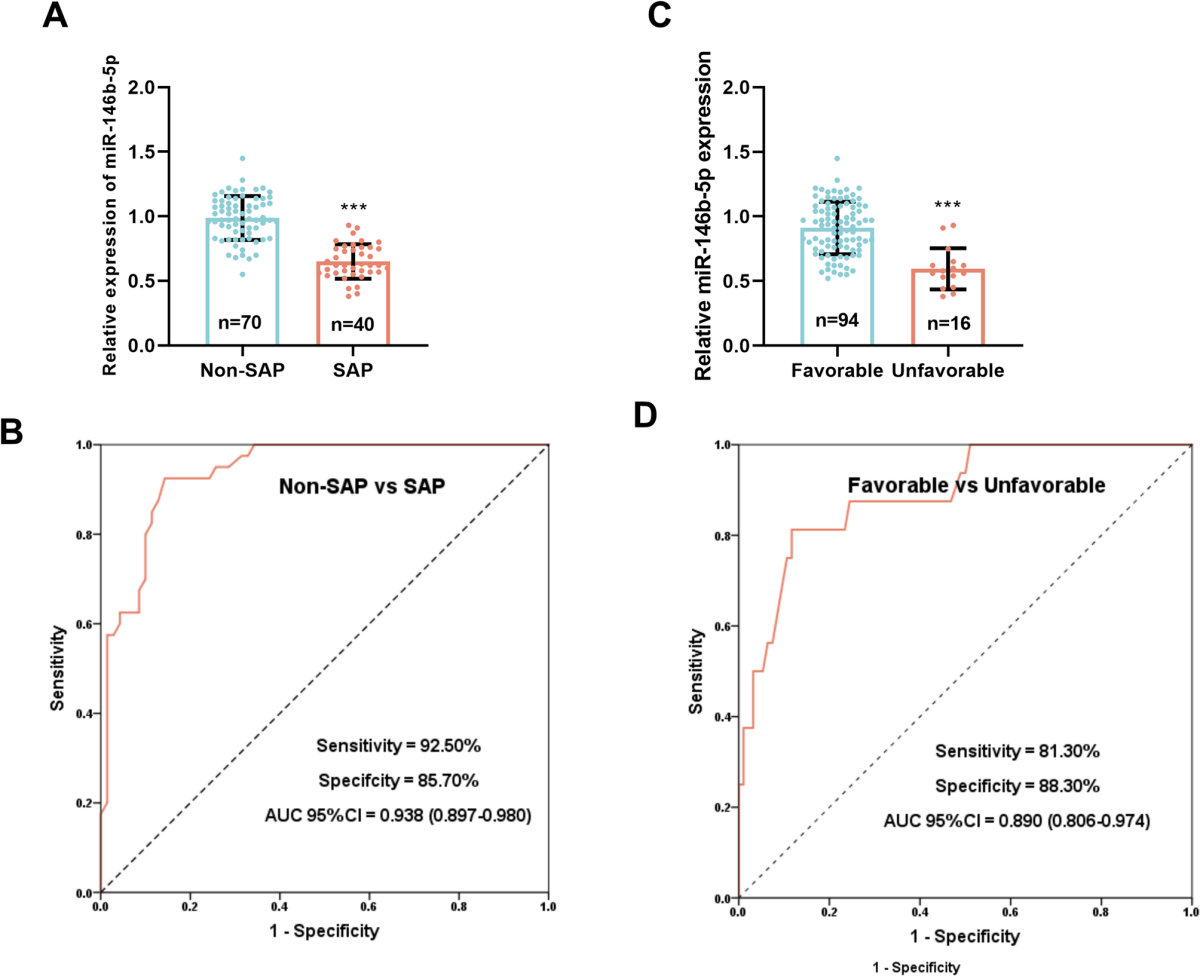
The diagnostic values of serum miR-146b-5p. (**A**). Serum miR-146b-5p in the non-SAP and SAP groups (^***^P < 0.001 vs. non-SAP group). (**B**). ROC curves were employed to assess the capability of miR-146b-5p to distinguish SAP patients from the non-SAP patients. (**C**). Serum miR-146b-5p levels in the favorable and unfavorable groups (^***^P < 0.001 vs. favorable group). (**D**). ROC curves were employed to assess the capability of miR-146b-5p to distinguish unfavorable patients from the favorable patients

To evaluate the role of miR-146b-5p in AP patients, serum miR-146b-5p was detected in all subjects by RT-qPCR, and then ROC curves were plotted. miR-146b-5p levels were lower in SAP patients compared with the non-SAP group (*P* < 0.001, Fig. 1A). AUC was 0.938, 95% CI was 0.897–0.980, sensitivity and specificity were 92.50% and 85.70% (Fig. 1B). Serum miR-146b-5p can distinguish SAP patients from non-SAP patients. Besides, it is shown in Fig. 1C that miR-146b-5p expression in the unfavorable group was significantly lower than that in the favorable group (*P* < 0.001). AUC was 0.890, 95% CI was 0.806–0.974, sensitivity and specificity were 81.30% and 88.30% (Fig. 1D). Serum miR-146b-5p can also distinguish unfavorable patients from favorable patients. This implies that serum miR-146b-5p is likely to serve as a significant and useful marker.

### miR-146b-5p level is negatively correlated with the severity of SAP

**Fig. 2:**
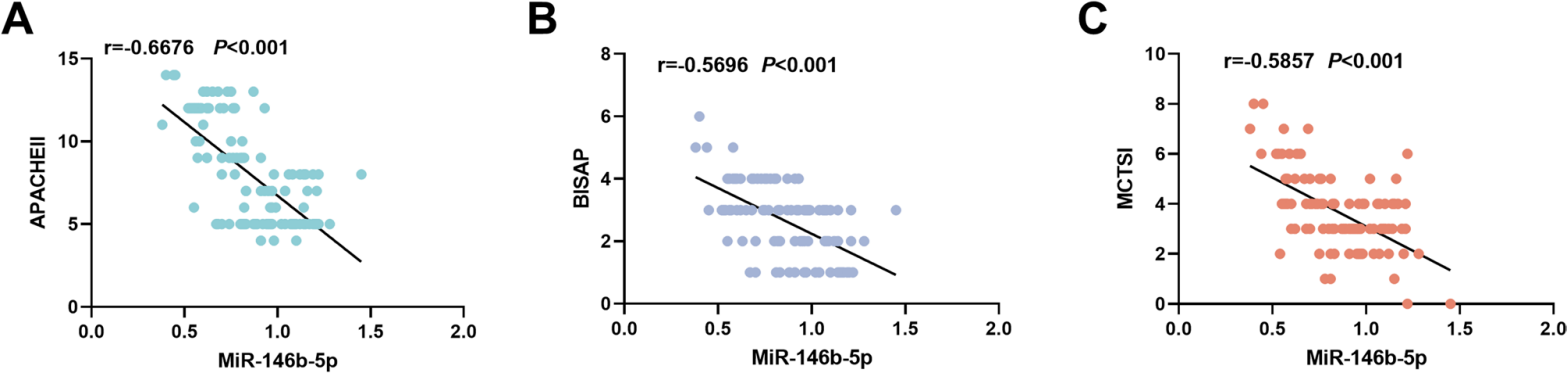
**C**orrelation between serum miR-146b-5p and APACHEII score (**A**), BISAP score (**B**) and MCTSI score (**C**) were evaluated by Pearson correlation coefficient

Pearson correlation coefficient was employed to examine the relationship among miR-146b-5p expression and the APACHEII score, BISAP score, as well as the MCTSI score. In AP patients, the serum miR-146b-5p exhibited an inverse correlation with APACHEII score (*r*=-0.6676, *P* < 0.001), BISAP score (*r*=-0.5696, *P* < 0.001), and MCTSI score (*r*=-0.5857, *P* < 0.001), as depicted in Fig. 2A-C.

### Impact of miR-146b-5p on the prognosis of SAP patients

Univariate analysis presented that serum miR-146b-5p (OR = 0.405, 95% CI = 0.178–0.922, *P* = 0.031), APACHEII score (OR = 2.222, 95% CI = 1.002–4.927, *P* = 0.049), BISAP score (OR = 2.821, 95% CI = 1.264–6.296, *P* = 0.011) and MCTSI score (OR = 2.333, 95% CI = 1.045–5.211, *P* = 0.039) indicators were the prognostic factors affecting SAP patients. In addition, multivariate analysis displayed that serum miR-146b-5p (OR = 0.316, 95% CI = 0.126–0.794, *P* = 0.014), BISAP score (OR = 2.689, 95% CI = 1.142–6.331, *P* = 0.024) and MCTSI score (OR = 2.537, 95% CI = 1.052–6.122, *P* = 0.038) were also independent prognostic factors influencing the poor prognosis of SAP patients (Table 2).

**Table 2:** Logistic regression analysis of clinical indicators for the occurrence of severe acute pancreatitis

Indicators	Univariate analysis		Multivariable analysis	
	OR (95%CI)	*P* value	OR (95%CI)	*P* value
Age (years)	0.958 (0.440–2.085)	0.914	/	/
Gender (male/female)	0.930 (0.424–2.038)	0.856	/	/
BMI (kg/m^2^)	1.357 (0.620–2.971)	0.445	/	/
Etiology	1.059 (0.686–1.636)	0.794	/	/
WBC (×10^9^/L)	1.239 (0.569–2.698)	0.589	/	/
HB (g/L)	0.931 (0.427–2.028)	0.857	/	/
HCT (%)	1.121 (0.515–2.440)	0.773	/	/
CRP (mmol/L)	1.207 (0.551–2.640)	0.638	/	/
IL-6 (pg/mL)	1.170 (0.538–2.547)	0.692	/	/
IL-8 (pg/mL)	0.773 (0.354–1.685)	0.517	/	/
APACHEII	2.222 (1.002–4.927	0.049	2.123(0.892–5.052)	0.089
BISAP	2.821 (1.264–6.296)	0.011	2.689 (1.142–6.331)	0.024
MCTSI	2.333(1.045–5.211)	0.039	2.537 (1.052–6.122)	0.038
miR-146b-5p	0.405 (0.178–0.922)	0.031	0.316 (0.126–0.794)	0.014

Abbreviations: WBC = white blood cell count; HB = hemoglobin; HCT = hematocrit; AMY = serum amylase; LPS = lipase; IL-6 = interleukin 6; CRP = C-reaction protein; APACHEII = acute physiology and chronic health evaluation II, MCTSI = Modified computed tomography severity index; BISAP = bedside index of severity in acute pancreatitis; *P* < 0.05 means significant difference

### miR-146b-5p alleviates damage in AR42J cells treated with DCA

**Fig. 3:**
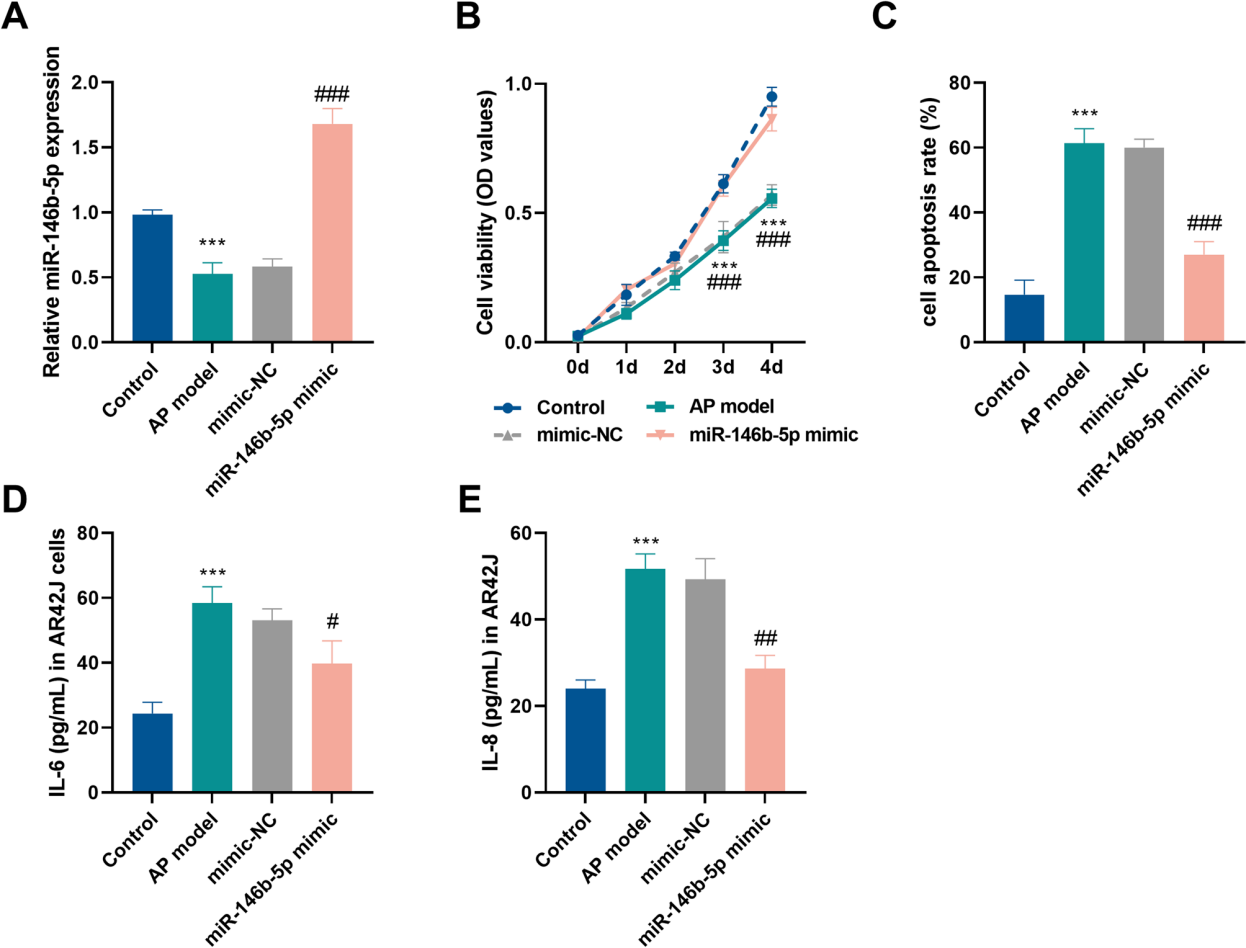
Effects of overexpression of miR-146b-5p on proliferation, apoptosis and inflammatory cytokines in DCA-treated AR42J cells. Effects of overexpression of miR-146b-5p in AR42J cells on miR-146b-5p expression (**A**), cell proliferation (**B**), apoptosis (**C**), IL-6 levels (**D**) and IL-8 levels (**E**). (^**^P < 0.01, ^***^P < 0.001 vs. control group, ^#^*P* < 0.05, ^##^*P* < 0.01, ^###^*P* < 0.001 vs. oe-NC group)

Compared with the control group, miR-146b-5p expression was reduced in AP model group. Notably, transfection of miR-146b-5p mimic could significantly improve the miR-146b-5p expression in the AP model group (*P* < 0.01, Fig. 3A). CCK-8 assays revealed a trend of time-dependent enhancement of cell viability in all groups. Cell viability was remarkably suppressed in the AP group at the third and fourth days compared to the control group. In addition, it was seen that up-regulation of miR-146b-5p clearly enhanced cell viability within AP group (*P* < 0.01, Fig. 3B). Flow cytometry data revealed that DCA induced massive death of AR42J cells. However, upregulation of miR-146b-5p diminished apoptosis (*P* < 0.001, Fig. 3C). DCA treatment induced a substantial elevation in IL-6 and IL-8, but overexpression of miR-146b-5p partially reversed this trend (*P* < 0.05, Fig. 3D-E).

## Discussion

Currently, the etiology of AP remains incompletely understood [24]. MAP is treatable, and patients can be discharged with a favorable prognosis. However, SAP may give rise to severe complications. In extreme cases, these complications may subsequently lead to organ failure and even result in death [25]. It is of particular importance for AP patients that early detection and treatment are carried out. Moreover, the selection of more suitable laboratory indicators might serve as the optimal predictors for the condition and prognosis of AP.

Serum miR-146b-5p was decreased in patients belonging to the SAP and unfavorable groups in this study. miR-146b-5p had a good diagnostic value. Additionally, miR-146b-5p levels were correlated with AP severity. miR-146b-5p, along with the APACHEII, BISAP, and MCTSI scores, served as prognostic factors for SAP patients. Further studies found that miR-146b-5p, BISAP score, and MCTSI score were independent risk factors for unfavorable prognosis in SAP patients. In the AP cell model treated with DCA, miR-146b-5p expression was reduced. The proliferative ability declined, and a significant number of cells entered apoptosis. However, when miR-146b-5p was overexpressed, this situation was partially reversed. Serum miR-146b-5p may be an indicator for the detection and prognosis prediction of AP.

In this study, quantitative analysis of OR values showed that for every one-unit reduction in serum miR-146b-5p in AP patients, the risk of poor prognosis increased by 2.16 times, confirming its significant association with poor prognosis. This indicator can be used as a supplement to existing prognostic assessment tools to more accurately identify high-risk patients. Compared with traditional biomarkers, amylase and lipase are rapidly elevated in the early stages of the disease, but have low specificity and weak correlation with disease severity and prognosis [26]. Sensitivity and specificity of CRP for diagnosing AP were 82% and 68% [27]. In contrast, miR-146b-5p in this study had a better diagnostic performance with a sensitivity of 92.50% and specificity of 85.70% in differentiating SAP from non-SAP patients. It has a sensitivity of 81.30% and specificity of 88.30% in distinguishing unfavorable from favorable patients, which is more advantageous than the reported IL-6 and IL-8 (sensitivity of 82% and 68%, specificity of 65% and 67%) [28]. In addition, miR-146b-5p level was significantly negatively correlated with AP disease severity, which is valuable for disease assessment, treatment adjustment and prognosis prediction. However, clinical translation faces technical and economic challenges: technically, RT-qPCR assays are insufficiently standardized, have high sample requirements, and the cost of assays and research and development limits their widespread use.

Previous studies have demonstrated the role of miR-146b-5p in various inflammatory and pancreas-related diseases [29]. Liu et al. confirmed by microarray analysis that miR-146b-5p was down-regulated in the serum of AP patients [13]. However, their study did not provide a detailed analysis of the diagnostic and prognostic value of miR-146b-5p in AP. In this study, we systematically integrated clinical data, serum miR-146b-5p levels, and multiple scoring systems. By employing cutting-edge biological techniques, we explored the mechanism of action of miR-146b-5p at the molecular and cellular levels and verified its down-regulation in SAP patients. Meanwhile, this study revealed that miR-146b-5p has the potential to serve as an early diagnostic and prognostic biomarker for AP. These findings provide a new perspective and basis for research in this field.

Much of the research on miR-146b-5p in the field of cancer has focused on its mechanism of action in tumorigenesis and progression [30]. For example, Lin et al. reported that miR-146b-5p effectively inhibited the migration and invasion of pancreatic cancer cells by targeting MMP16 [31]. In the present study, by constructing an AP cell model, it was demonstrated that down-regulation of miR-146b-5p expression leads to diminished cell proliferation and apoptosis in a large number of cells. When miR-146b-5p expression was up-regulated, cell proliferation was enhanced and apoptosis was inhibited. This indicates that miR-146b-5p might be significantly involved in the initiation and development of AP by controlling critical aspects of cell proliferation and apoptosis.

With the intensification of research on AP, the role of inflammatory cytokines in AP has increasingly drawn attention. Particularly in the case of SAP, a variety of inflammatory factors are elevated in the pancreas, as well as in distant organs, tissues, and cells [32, 33]. This may be due to the activation of pancreatic proteases and the impaired pancreatic microcirculation in AP, which stimulate the secretion of cytokines such as IL-6 from granulocytes, macrophages and vascular endothelial cells [34]. Under other inflammatory conditions, miR-146b-5p has been shown to have anti-inflammatory effects [35]. For example, in lupus nephritis (LN), serum miR-146b-5p was reduced in lipopolysaccharide-induced patients and LN models, suggesting a role in modulating the inflammatory response [36]. Similarly, in clear cell renal cell carcinoma, miR-146b-5p promoted tumor growth by inhibiting SEMA3G, further highlighting its dual role in cancer and inflammation [37]. In the present study, we constructed a cellular model for AP and observed that up-regulation of miR-146b-5p resulted in a decrease in IL-6 and IL-8 protein levels, a result that is highly consistent with the anti-inflammatory properties of miR-146b-5p. This offers more evidence for miR-146b-5p’s potential to be a therapeutic goal for AP.

At present, the mechanism by which miR-146b-5p functions in AP is not completely clear and requires further study. Referring to studies related to miRNA regulation of disease processes, e.g., Panagal et al. found that miR-21 can affect stroke neuronal cell survival and apoptosis by regulating specific target genes [38]. It was hypothesized that miR-146b-5p may be involved in AP pathology in a similar mode. In cell proliferation, miR-146b-5p may bind to the 3’-UTR of cell cycle-related genes, inhibiting mRNA translation or accelerating its degradation, interfering with the cell cycle and reducing proliferation [39]. In the regulation of apoptosis, it may target genes such as Bcl-2 family and caspases to affect the balance between proliferation and apoptosis of pancreatic cells in AP [40]. In addition, NF-κB, as a key regulator of the inflammatory response, promotes the release of inflammatory factors, exacerbates the inflammatory response and pancreatic tissue injury, and miR-146b-5p is highly likely to be involved in regulating the NF-κB signaling pathway [41]. It has been shown that miRNAs play key regulatory roles in complex signaling networks, e.g., Selvakumar et al. found that miRNAs can influence tumor development through the PTEN/PI3K/AKT pathway [42]. Notably, in alveolar epithelial cells, MINCR affects TRAF6 and NF-κB pathways via regulation of miR-146b-5p, triggering injury and inflammation [43]. In AP-induced intestinal barrier injury, TRAF6 activates the TLR4/NF-κB pathway to exacerbate the injury, and inhibition of its expression attenuates inflammation [44]. Based on this speculation, miR-146b-5p may be involved in the inflammatory response of AP by targeting TRAF6 and NF-kB pathways, but the specific mechanism of action will be explored in future studies.

There are some limitations of this study. Firstly, the relatively small sample size of the SAP subgroup, the single source, and the homogeneity of patients may affect the generalizability of the results. Secondly, the mechanistic studies are not deep enough and lack systematic analysis of its downstream target genes and signaling pathways. Thirdly, the research model is relatively simple, only conducting functional verification from the in vitro cellular level. Moreover, focusing only on IL-6 and IL-8 as inflammatory markers due to their importance and feasibility, while omitting other cytokines, weakens the study’s robustness. Future research will be deepened in multiple dimensions: first, the sample size will be expanded, and more accurate diagnostic and prognostic models will be constructed. Second, multiple experimental techniques will be used to analyze the downstream targets and signaling pathways and mechanisms of action of miR-146b-5p. Third, based on reports from previous studies, this study will explore the potential application of miR-146b-5p-targeted therapy in combination with preventive strategies such as vitamin D supplementation [45]. Fourth, incorporating more AP-related cytokines, such as TNF-α, IL-10, and IFN-γ, to better understand inflammation. In addition, it is planned to construct an animal model of AP to verify its regulatory role on the disease process at the in vivo level and provide more sufficient evidence to reveal the pathogenesis of AP.

Despite the limitations of the study, miR-146b-5p remains highly promising for AP clinical applications. In terms of diagnosis, its serum test is simple and cost-controlled, and its combination with traditional methods can significantly improve the accuracy of early diagnosis of AP. During prognostic assessment, it can be used as a key indicator to assist physicians in optimizing treatment plans and improving patient prognosis. The therapeutic area holds promise for the development of innovative therapies based on its protective effects on AP cell models.

In conclusion, the results suggest that serum miR-146b-5p is reduced in SAP patients and is strongly linked to disease severity and poor prognosis. The upregulation of miR-146b-5p leads to an enhancement in cell proliferation, a suppression of apoptosis, and a decline in the production of IL-6 and IL-8 proteins. In addition, in the context of nursing, serum miR-146b-5p is dysregulated in patients with AP, which may suggest that an inflammatory response is occurring in the patient’s body. Treatment can be carried out under the guidance of a doctor. MAP patients follow the diet prescribed by the doctor, avoid overeating and drinking alcohol, and encourage moderate activity. SAP patients are treated with somatostatin and other therapies under the guidance of doctors. Meanwhile, vital signs and inflammatory indicators need to be closely monitored to adjust the scheme, pay attention to the contraindications and adverse reactions when using the drug to ensure the effectiveness of the treatment for patients.

## Electronic supplementary material

Below is the link to the electronic supplementary material.

Supplementary Material 1

## Data Availability

The datasets used and analysed during the current study are available from the corresponding author on reasonable request.
