# Supplementary material for: Diagnostic and prognostic value of miR-146b-5p in acute pancreatitis
Source: Hereditas. 2025 May 31;162:93. doi: 10.1186/s41065-025-00466-9 (PMC12126874; doi:10.1186/s41065-025-00466-9)
Supplement: Supplementary file 1 — Supplementary Material 1 [file 41065_2025_466_MOESM1_ESM.docx]

**Table S1** List of abbreviations

| ANOVA | Analysis of variance |
| --- | --- |
| AP | Acute pancreatitis |
| APACHEII | Acute Physiology and Chronic Health Evaluation II |
| AUC | Area under the curve |
| BISAP | Bedside Index of Severity in Acute Pancreatitis |
| BMI | Body mass index |
| CCK-8 | Cell Counting Kit-8 |
| CI | Confidence interval |
| CRP | C-reaction protein |
| DCA | Deoxycholic acid |
| ELISA | Enzyme-Linked Immunosorbent Assay |
| FBS | Fetal Bovine Serum |
| HB | Hemoglobin |
| HCT | Hematocrit |
| IFN-γ | Interferon-γ |
| IL-6 | Interleukin 6 |
| IL-8 | Interleukin 8 |
| IL-10 | Interleukin 10 |
| MCTSI | Modified Computed Tomography Severity Index |
| MAP | Mild acute pancreatitis |
| OF | Organ failure |
| OR | Odds Ratio |
| ROC | Receiver operating characteristic |
| SAP | Severe acute pancreatitis |
| SD | Standard deviation |
| TNF-α | Tumor Necrosis Factor-α |
| WBC | White blood cell count |
